# The effect of Er, Cr:YSGG laser debonding on the bond strength of two ceramic materials to dentin

**DOI:** 10.1186/s12903-023-02721-9

**Published:** 2023-01-12

**Authors:** Hoda M. Abdel Sadek, Ahmed M. Abdel Khalek, Marwa M. Wahsh

**Affiliations:** 1 Department of Fixed Prosthodontics, Faculty of Dentistry, Ain Shams University, Organization of African Unity St, El-Qobba Bridge, El Weili, Cairo, Egypt; 2 Department of Fixed Prosthodontics, Faculty of Dentistry, Galala University, Ain Shams University, Cairo, Egypt; 3 General Organization of Teaching Hospitals and Institues, Cairo, Egypt

**Keywords:** Laser debonding, Resin cement, Shear bond strength, Er,Cr:YSGG laser, Ceramics

## Abstract

**Objectives:**

Evaluating the bond strength of two ceramic materials to dentin after Er,Cr:YSGG laser debonding. Would laser debonding affect the bond strength of ceramic to dentin?

**Materials and methods:**

Recently extracted human molars were ground to expose dentin. Forty square shaped samples were prepared from CAD/CAM ceramic blocks. Samples were divided into two groups according to the type of ceramic material; group E: Lithium disilicate and group T: Ultra-translucent Zirconia (n = 20) Each group was divided into two subgroups (n = 10) according to the laser debonding effect (subgroup B: bonded samples, subgroup R: re-bonded samples after laser debonding). Ceramic samples were bonded to dentin using dual cure self-adhesive resin cement. Laser debonding of ceramic samples of subgroups R using Er, Cr:YSGG laser, were then re-bonded again to dentin surface with same resin cement. The Shear bond strength test using Universal testing machine was done. The failure mode was analyzed. Two-way analysis of variance was used to compare the mean bond strength and re-bond strength of two materials. The significance level was set at *P* ≤ 0.05.

**Results:**

Two-Way ANOVA showed that ceramic type had a significant effect on the re-bond strength to dentin. The predominant failure mode was adhesive.

**Conclusions:**

Laser debonding of Lithium disilicate and Ultra translucent Zirconia decreased the re-bond strength to dentin. Deterioration in re-bond strength for Lithium disilicate ceramics was more pronounced than for Ultra translucent Zirconia. *Clinical Relevance* Deterioration in the bond strength between ceramics & dentin after laser debonding still needs improvement to allow its clinical use.

## Introduction

All-ceramic restorations are widely spread nowadays helping dentists to fulfill the increasing esthetic demands of the patients as well as restoring function. These materials introduced many advantages superior to old metal or porcelain fused to metal PFM restorations as tooth colored natural appearance gives optimum esthetics and high strength which is close to natural teeth. Besides that, using resin cements as luting agents which bond chemically to both tooth structure and most of all-ceramic restorations achieving a Monoblock allowing better stress distribution and longevity of restorations and remaining tooth structure [1].

Many types of all-ceramic materials have been introduced to the market which include lithium disilicate glass ceramics, hybrid ceramics, Partially stabilize Zirconia, ultra-translucent Zirconia and Zirconia reinforced glass ceramics [2]. Lithium disilicate is composed of quartz, lithium dioxide, phosphor oxide, alumina, potassium oxide, and other components. It has excellent esthetics and high strength with versatile applications and extensive indication range. Alongside that, it has natural-looking esthetics irrespective of the preparation shade [3]. Moreover, the minimally invasive preparation and adhesive cementation of lithium disilicate restorations with thickness of 1 mm provide long term clinical success with scientifically documented results [3–5].

Zirconia (ZrO_2_) is type of ceramics which used as partial or full coverage monolithic restoration to optimize shape, function, and color of restoration. Recently, more translucent monolithic ZrO_2_ has been introduced for anterior and posterior restorations. The traditional 3-mol % yttria (3-mol%Y) ZrO_2_ combines high strength but relatively poor translucency [6]. However, ZrO_2_ materials contain higher Y is more translucent, having the advantage of being much more esthetic due to their higher translucency but have a disadvantage of a reduction the mechanical properties [7].

The biggest challenge which faces the clinicians when using all ceramic restoration is the difficulty to retrieve to manage complications due to the hardness of the material and higher bond strength of resin cement [8].The resin cements are difficult to distinguish from tooth structure as they are clear or tooth-colored, so crown removal can be done with many rotary cutting instruments which consume much chair time [9]. The sacrifice of the restoration is the safest and least traumatic method of removal as a slot is cut buccolingually through the crown or retainer to separate it into two halves then the segments are separated with a rigid instrument. However, several factors may necessitate intact removal and re-cementation of the restoration. These factors include the patient’s age and health, the time involved, esthetics, and financial considerations as well as social and psychological concerns [10].

Recently, with the technological advance in the dental field, laser applications increased dramatically in the last few years for both hard and soft tissues. Using laser for debonding was first used for debonding ceramic orthodontic brackets and has been experimentally used since 1990s for this procedure. With the laser-based technique, debonding occurs within 1 to 5 s and does not cause patient discomfort or irreversible pulpal changes [11]. Lasers such as erbium: yttrium aluminum- garnet (Er:YAG) and erbium, chromium: yttrium-scandium-gallium garnet (Er,Cr:YSGG), have been used to remove restorative materials, including laminate veneers and crowns [8, 12, 13]. The effect of laser debonding on bond strength of resin cement to ceramic was not tested in the literature but the re-bond strength was tested after using (Er,Cr:YSGG) laser to remove the remnants of bonding materials from base of failed ceramic orthodontic brackets, which was effective [14]. A strong and durable resin-ceramic bond improves marginal adaptation and provides high retention [15]. The surface alterations may occur on the ceramic surface due to laser irradiation as it causes changes in surface roughness [16, 17]. The main effect of the laser energy is the conversion of light energy into heat, so the significant effect between the laser and substrate when increase the absorption of the laser energy by the substrate. In addition to other surface qualities, pigmentation of the surface and its water content determines the extent of energy absorbed by the irradiated surface [12]. Therefore, the aim of this study was to evaluate the bond strength of two ceramic materials after Er,Cr:YSGG laser debonding. The null hypothesis was that there will be no significant difference between the bond strength and re-bond strength after Er,Cr:YSGG laser debonding of the two ceramic materials.

## Materials and methods

The material used in this study, trade name, manufacturer, composition, and lot number listed in Table 1.

**Table 1:** The description of materials used in study

Description	Trade name	Composition	Manufacturer	LOT number
Lithium disilicate glass ceramics	IPS e.max CAD	40% lithium metasilicate crystals (Li2SiO3), set in a glassy phase	Ivoclar Vivadent AG, Schaan, Liechtenstein	X54892
Ultra-translucent Zirconia	BruxZir™ Anterior Milling Blanks	5 Y-TZP zirconia contains 5 mol %yttria. partially stabilized with approximately 50% cubic zirconia	Glidewell Dental Laboratory, California, USA	BZ0009409
Hydrofluoric Acid	DentoBond Porcelain Etch	8% Hydrofluoric Acid, 90.5% Aqua 1.5% Xanthan gum	Itena clinical, Paris, France	4178-21PFXE
Silane coupling agent	DentoBond Porcelain Silane	97% Ethyl Alcohol, 3%Glycidoxypropyltrimethoxysilane	Itena clinical, Paris, France	4185-21PFXS
Zirconia primer	Z-Prime plus	Ethanol, BisGMA, 2-Hydroxyethyl Methacrylate, MDP	Bisco, Schaumburg, USA	1900006919
Dual-cured Self-adhesive resin cement	TOTALCEM	UDMA, Bis-GMA, TEGDMA, 4-META	Itena clinical, Paris, France	4256-42HQBSETR

### Tooth selection and preparation

**Fig. 1:**
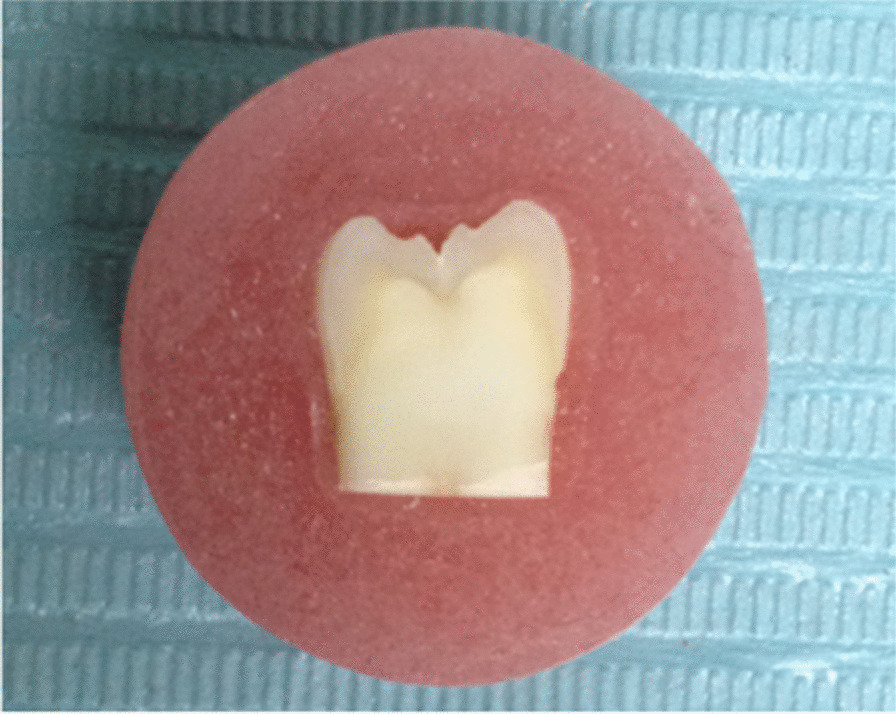
Ground tooth imbedded in acrylic resin block

Sound freshly extracted mandibular molars free of carious lesions and cracks were selected for this study [Ethics committee approval: FDAsu-RecEM041810]. All external debris were removed with an ultrasonic scaler and teeth were stored in distilled water for one month before used in study. Teeth were sectioned into two halves and buccal surfaces were ground to expose dentin using low speed precision saw (IsoMet™ 4000, BUEHLER. USA) to provide a flat surface for bonding. The roots of teeth were cut off 2 mm below CEJ. Teeth were fixed in mold filled with acrylic resin (Fig. 1), then stored in distilled water at room temperature until used.

### Samples grouping

A power analysis was designed to have adequate power to apply a statistical test of the null hypothesis that no difference would be found between tested groups. By adopting an alpha (α) level of (0.05), a beta (β) of (0.2) (i.e., power = 80%), and an effect size (f) of (0.569) calculated based on the results of a previous study [18]; the predicted sample size (n) was found to be (10) samples.

A total of 40 square shaped samples (4 × 4 mm and 1 mm thickness resembling crown thickness) were prepared from CAD/CAM (computer aided design/computer aided manufacture) ceramic blocks. Samples were divided into two groups (n = 20) according to type of ceramic material, group E: Lithium disilicate (IPS e.max CAD, Ivoclar Vivadent, Schaan, Liechtenstein), group T: Ultra-Translucent Zirconia (BruxZir®, Glidewell Dental Laboratory, California, USA). Each group was divided into two subgroups (n = 10) according to laser debonding effect (subgroup B: bonded samples, subgroup R: re-bonded samples after laser debonding.

### Samples fabrication and bonding procedures

Samples of group (E) lithium disilicate glazed and crystallized in ceramic furnace (Ivoclar viva dent, Liechtenstein, Germany) according to manufacturer recommendations, then allowed to cool in room temperature. Samples of group (T) ultra-translucent zirconia were sintered and glazed in zirconia furnace (Nabertherm, Germany) according to the manufacturer’s recommendations.

### Ceramic surface treatment

Lithium disilicate samples were etched by HF acid (DentoBond Porcelain etch, ITENA, Paris, France.) for 20 s then the samples were rinsed thoroughly with water for 60 s to completely remove the etchant and dry well, followed by the application of silane on ceramic samples (DentoBond Porcelain silane, ITENA, Paris, France) for 60 s according to manufacture instructions. While Ultra-translucent zirconia samples were air abraded by aluminum oxide particles size 110 micron at 1.5 bar and 2 mm distance, Zirconia primer (Z prime plus zirconia primer, BISCO, Schaumburg, USA) were then applied.

### Bonding

Bonding of ceramic samples was performed to exposed dentin on prepared samples’ surfaces using dual curing self-adhesive resin cement (TOTALCEM by ITENA medical, Paris, France). A flat blunt back of hand instrument was used to apply pressure to produce uniform film thickness of cement and for samples fixation [19]. The Light cure (LED.F, WOODPECKER®), its wavelength (420–480 nm), used in high power mode. Short initial light curing or "tack curing" for 3 s was done to create a semi-gel state in luting cements for easier excess cement cleanup [20], then excess cement was carefully removed at the margins using sharp explorer. Curing was continued for 20 s at high power mode.

### Laser debonding

**Fig. 2:**
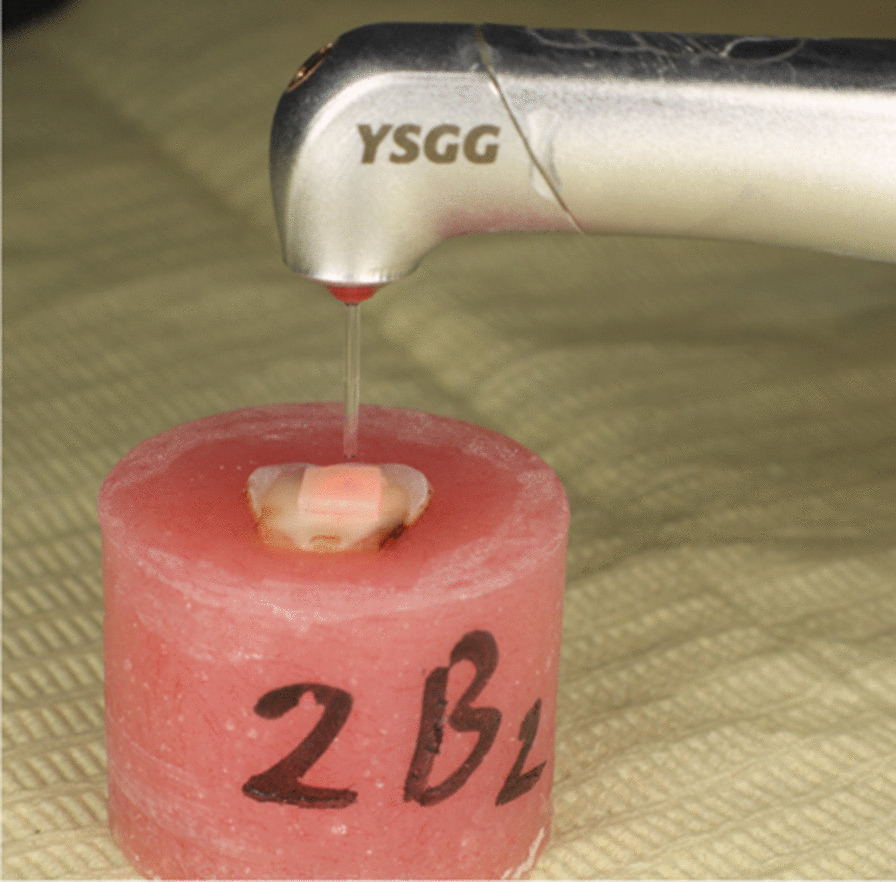
LASER application perpendicular to ceramic surface

Samples of subgroups (R) were de-bonded from dentin using Er,Cr:YSGG laser (Biolase Waterlase iPlus 2.0,USA) [8], wave length 2780 nm, according to the following settings; H-mode, average power: 6 W, frequency: 20 Hz, Pulse duration 60 microseconds, 80%Water, 60%Air. Er,Cr:YSGG: Gold Hand piece was selected for the study using MGG6 Saffire tip 600 µm diameter, positioned perpendicular to ceramic sample surface at a distance 2 mm (Fig. 2), energy applied by scanning method through surface for 10 s [21] with horizontal movements perpendicular to the surface, and debonding was checked using sharp explorer every 5 s until debonding.

### Cleaning and re-bonding

The Teeth were stored in distilled water after de-bond for 1 h before re-bond. Fitting surfaces of all de-bonded samples of subgroups (R) of both lithium disilicate and Ultra translucent zirconia were checked for cement remnants which was removed by sandblasting with 50-micron aluminum oxide at 1 bar (15 psi) pressure for 20 s [22], then re-bonded to dentin surface after refreshment and removal of remnants of cement with diamond finishing stone by the same protocol done for bonding according to the manufacturer’s recommendations.

### Shear bond strength test (SBS)

**Fig. 3:**
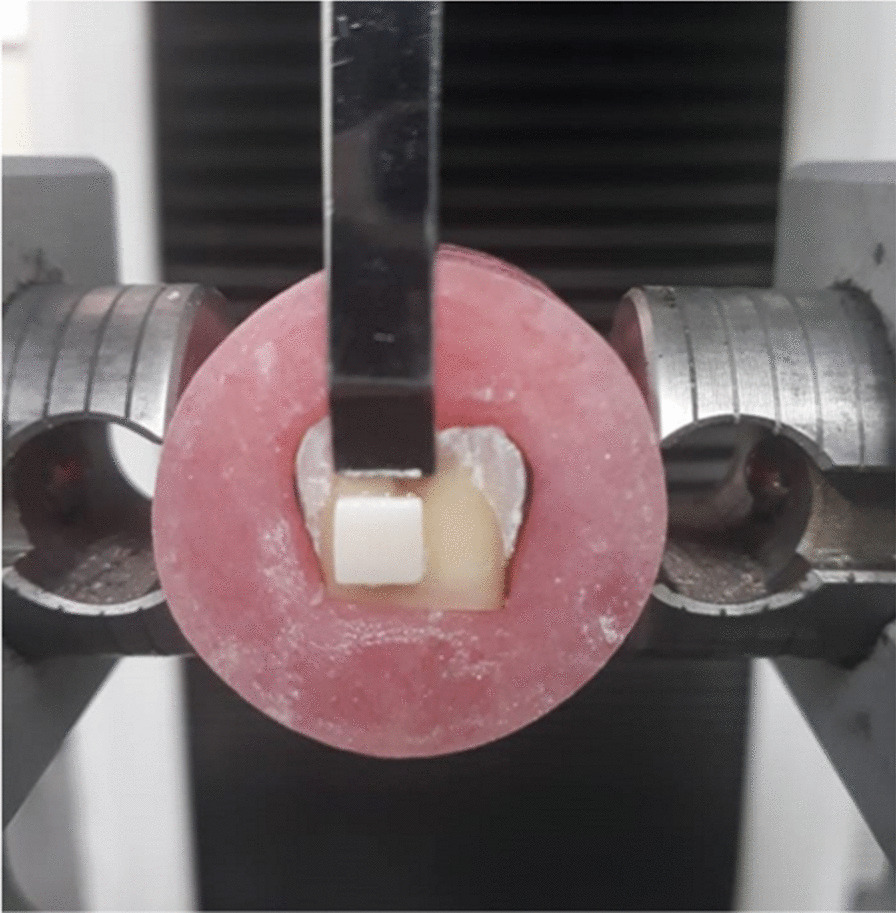
Measuring shear bond strength using universal testing machine

Shear bond strength test [23] was employed on all samples of both subgroups B&R of both lithium disilicate and Ultra translucent Zirconia using universal testing machine (model 3345, England) (Fig. 3). The data was calculated and recorded using computer software (Bluehill, Instron, England). Numerical data was explored for normality by checking the data distribution and using Shapiro-Wilk tests. Data showed the parametric distribution; they were represented by mean and standard deviation (SD) values. Two-way ANOVA was used to study the effect of different tested variables and their interaction. Comparison of the main and simple effects was done by utilizing pairwise t-tests with Bonferroni correction. The significance level was set at *p* ≤ 0.05 within all tests. Statistical analysis was performed with SPSS Version 26 for Windows (SPSS, Inc., IBM Corporation, NY, USA).

### Evaluation of failure pattern

All ceramic samples fitting surfaces were checked under digital microscope (Nikon, 50X, Ma100, JAPAN), at 30 × magnification. The failure mode was analyzed as adhesive, cohesive and mixed failures.

## Results

Mean and standard deviation values of shear bond strength for all groups were presented in Table 2. Two-Way ANOVA showed that ceramic material and laser debonding had significant effect on the shear bond strength. The interactions between the independent variables, ceramic material, and laser debonding had significant effect on the shear bond strength.

**Table 2:** Mean ± standard deviation (SD) of shear bond strength (MPa) for two ceramic materials and laser debonding status

Shear bond strength (mean ± SD)				*p*-value
Lithium disilicate -Bond	Lithium disilicate -Rebond	Ultra-translucent zirconia-Bond	Ultra-translucent zirconia-Rebond	
6.53 ± 1.60^a^	1.12 ± 0.05^c^	6.43 ± 2.28^a^	4.33 ± 1.88^b^	< 0.001*

Means with different letters are statistically significant, *significant (*p* ≤ 0.05)

One-way ANOVA was followed by Tukey’s post hoc test (Table 2) showed that the highest value was found in Lithium disilicate -Bond group, while the lowest value was found in Lithium disilicate-Rebond. Post hoc pairwise comparisons showed Lithium disilicate -Rebond group to have a significantly lowest value than other groups (*p* < 0.001).

**Fig. 4:**
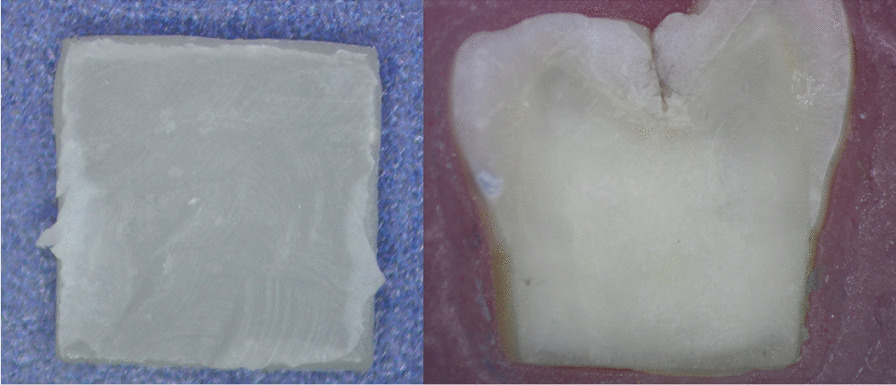
Adhesive failure in lithium disilicate re-bond group

**Fig. 5:**
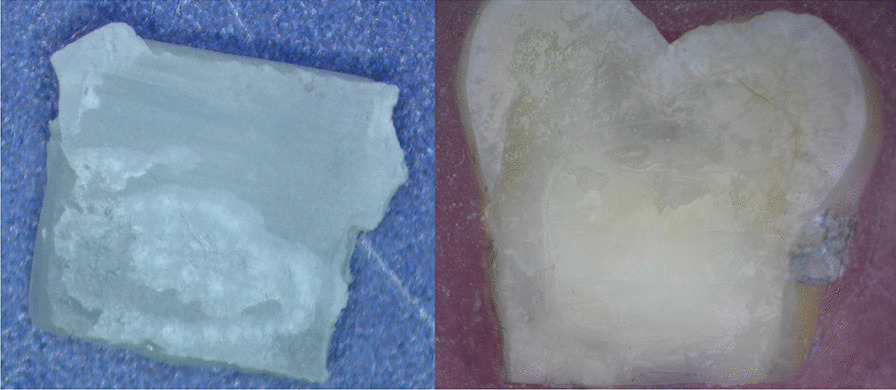
Mixed failure in ultra-translucent zirconia bond group

The failure mode analysis among all the experimental groups showed in (Table 3). There was no complete cohesive failure either in ceramic or resin cement (0%). The predominant failure type was adhesive failure 75% between cement and dentin while the remaining 25% was mixed failure between cement and dentin (Figs. 4 and 5).

**Table 3:** Number of samples from each group for each type of failure mode

Failure mode	Cohesion failure	Adhesive failure	Mixed failure	Total
Groups				
EB	0	6	4	10
ER	0	7	3	10
TB	0	8	2	10
TR	0	8	2	10
Total	0	29	11	40

## Discussion

Indirect adhesive all ceramic restorations are considered a treatment for mutilated teeth using crowns, endocrowns, inlays and onlays, as well as for tooth structure preservation such as veneers which is vastly used in daily practice. The demand for sound retrieval of these restorations in many clinical conditions is increased which is considered a big challenge for the clinicians. This process was not applicable with the available instruments and tools, pushing the clinicians to damage all ceramic restoration intentionally which is a time-consuming procedure [9, 13]. The invention of hard tissue laser and its properties in reacting with ceramics and resins allowed its use to remove all ceramic restorations in sound condition, many studies on removal of all ceramic by laser were done to establish evidence based practice from all aspects. [24]

There is little data in literature regarding bond strength of ceramic materials to tooth structure after laser debonding. This study evaluated the effect of debonding of two wide spread ceramic materials (lithium disilicate ceramics and Ultra translucent Zirconia by Er, Cr:YSGG laser on the bond strength to dentin after re-bonding. Laser debonding of ceramic restorations are mostly performed using Er:YAG (2940 nm) and Er,Cr:YSGG (2780 nm) laser. Short-pulsed laser ablation is a promising method for the debonding of all ceramic restorations while avoiding overheating of the pulp as if the cement is rapidly ablated, then heat conduction by the slow thermal softening process can be avoided [25]. Rising pulse repetition rate during resin removal results in a linear increase in the pulpal temperature, but still does not cause a temperature increase above the safe limit for the pulp vitality [26]**.** These considerations were considered in this study by selecting a high-power setting of 6 W and a frequency (Repetition Rate) of 20 Hz, which resulted in an increase in energy per pulse and a decrease in pulse duration, so that the cement is rapidly ablated to avoid heat conduction by a slow thermal softening process.

The results of the current study showed that ceramic material and laser debonding had statistically significant effect on the shear bond strength, so the null hypothesis was rejected. The effect of Er,Cr:YSGG laser debonding on the re-bond strength of the materials used showed that there was a significant difference in re-bond SBS for both ceramic materials lithium disilicate and ultra-translucent zirconia, re-bond strength mean values were 1.12 ± 0.05 MPa for IPS e.max CAD and 4.08 ± 1.88 MPa for the BruxZir group, and there was statistical difference between the two re-bond groups. By comparing the SBS results of bond groups and re-bond groups, lithium disilicate re-bond group has shown large decrease in bond strength after laser application, as the mean SBS was in bond group 6.54 ± 1.59 MPa and 1.12 ± 0.05 MPa for re-bond group of the same ceramic material. While in ultra-translucent zirconia bond group the mean SBS was 6.43 ± 2.28 MPa, and 4.08 ± 1.88 MPa for the re-bond group showing significant decrease in re-bond strength.

This significant decrease in re-bond strength under the limitation of this study may be due to changes in dentin surface after Er,Cr:YSGG laser application for debonding ceramic samples, as smear layer presence affected by laser time application as mentioned by Mahdisiar et al. [27], and structural changes in dentinal tubules found by Wang et al. [28], where the surface morphology of the dentin changed after Er:YAG laser pre-treatment with different energy and frequency values. The dentinal tubules opened within a specific energy (50–200 mJ) and frequency (5–20 Hz) range. Beyond this range, the inter-tubular dentin showed cracks and structural disintegration.

The re-bond strength of IPS E.max CAD showed deterioration in bond strength more than that for BruxZir anterior, this can be explained by difference in laser transmission through the different ceramic material because of different composition between lithium disilicate and ultra-translucent Zirconia ceramics, laser transmission through lithium disilicate is higher [29] as polycrystalline nature of ceramics hinder laser transmission through the material [30] so delivering lower energy through resin and dentin.

In this study the mean of shear bond strength (SBS) values in Bond groups (without laser application) in lithium disilicate IPS e.max CAD and the ultra-translucent Zirconia BruxZir anterior were 6.54 ± 1.59 MPa and was 6.43 ± 2.28 MPa respectively, and there was no statistical significance between them. This may be as the failure mode was most commonly adhesive with dentin due to use of self-adhesive resin cement which has highest bond strength in ceramic / resin interface when treating ceramic surface with hydrofluoric acid and silane coupling agent while lower bond strength in resin / dentin interface as no pre-treatment of dentin surface was done as manufacturer recommendations [31]. This is supported by earlier studies done by Wang et al. [28], Hattar et al. [32] and Malysa et al. [33]. The self-adhesive resin cements bonded weaker to dentin than bonding to ceramic material both lithium disilicate and Zirconia, and this explains the low values of bond strength in bond groups of this study. The lower shear bond strength of the self-adhesive resin cements may be due to the fact that this type of material interacts superficially with mineralized tissue and can’t demineralize the dentin completely. Thus, the smear layer cannot be completely removed, and hence, it is impossible to achieve the full formation of resin tags in the hybrid layer [34]. This was similar to results of study by Hattar et al. [32], as bond strength value to dentin recorded was 5.94 ± 2.17 MPa while evaluating three different self-adhesive resin cements to enamel and dentin. In another study by Malysa et al. [33] a higher shear bond strength values were recorded, and this may be due to using different resin cements, beside using smaller diameter samples, as surface area inversely proportional with bond strength [34, 35], the larger the surface area the lower shear bond strength will be. Beside that the condition of dentin whether dry or moist affected bond strength of resin cements as mentioned by André et al. [36], as bonding to moist dentin enhanced bond strength, this may be variation in this study which affected bond strength values.

Limitations of this study were using one type of laser with one set of parameters, using one type of resin cement, no aging were done for the samples and not studying full contour crowns.

Further research is still need prior to clinical trials to improve the bond strength of ceramics to tooth structure after laser debonding due to the deterioration of bond strength.

## Conclusion

Under the used parameters in the study and limitations the following conclusions could be drawn:

1. The use of Er,Cr:YSGG laser for debonding lithium disilicate and ultra-translucent zirconia decreased the re-bond strength.
2. Deterioration in re-bond strength for Lithium disilicate ceramic was more pronounced than for Ultra translucent Zirconia.
3. The most frequent type of failure in bond was adhesive at dentin cement interface.

### Recommendations for future studies

It is preferable to use a different type of resin cement and to treat the dentin differently before re-bonding. Furthermore, changing the setting parameter of the Er,Cr:YSGG laser for dependability might alter re-bond strength.

## Data Availability

The datasets used and analyzed during the current study available from the corresponding author on reasonable request.
